# Silver Nanoparticles Loaded with Oleuropein Reduce Doxorubicin-Induced Testicular Damage by Regulating Endoplasmic Reticulum Stress, and Apoptosis

**DOI:** 10.1007/s12011-024-04058-y

**Published:** 2024-01-10

**Authors:** Elif Erbaş, Volkan Gelen, Hülya Kara, Semin Gedikli, Ali Yeşildağ, Seçkin Özkanlar, Serkan Ali Akarsu

**Affiliations:** 1 Department of Histology and Embryology, Faculty of Veterinary Medicine, Atatürk University, Erzurum, Turkey; 2 Department of Physiology, Faculty of Veterinary Medicine, Kafkas University, Kars, Turkey; 3 Department of Anatomy, Faculty of Veterinary Medicine, Atatürk University, Erzurum, Turkey; 4 Department of Bioengineering, Faculty of Engineering and Architecture, Kafkas University, Kars, Turkey; 5 Department of Biochemistry, Faculty of Veterinary Medicine, Atatürk University, Erzurum, Turkey; 6 Department of Reproduction and Artificial Insemination, Faculty of Veterinary Medicine, Ataturk University, Erzurum, Turkey

**Keywords:** Silver nanoparticles, Oleuropein, Apoptosis, Inflammation, Endoplasmic reticulum-stress

## Abstract

**Abstract:**

Doxorubicin (DOX) is the most used chemotherapeutic agent for treating solid tumors. DOX treatment may lead to testicular damage using oxidative stress, resulting in infertility. These adverse effects may be prevented by the activation of antioxidant systems.

Oleuropein (OLE) is a powerful flavonoid with several ameliorative effects, including antioxidative, antiproliferative, and anti-inflammatory. It would be more efficient and applicable in treating chronic human diseases if its poor bioavailability improves with a nano-delivery system. The current study aims to assess the histopathological changes and antioxidative effects of OLE loaded with silver nanoparticles oleuropein (OLE-AgNP) on the testicular injury triggered by DOX in rats.

Forty-eight male albino rats were randomly divided into six groups as follows: the control, DOX (2.5 mg/kg), OLE (50 mg/kg), AgNP (100 mg/kg), OLE + AgNP (50 mg/kg), OLE (50 mg/kg) + DOX (2.5 mg/kg), AgNP (100 mg/kg) + DOX (2.5 mg/kg), and OLE-AgNP (50 mg/kg) + DOX (2.5 mg/kg) for 11 days. Oxidative stress, inflammation, apoptosis, endoplasmic reticulum stress markers, sperm analysis, and histopathological analyses were performed on testicular tissues taken from rats decapitated after the applications and compared between the experimental groups. The tissue MDA level was lower in the OLE and OLE+AgNP-treated groups than in the DOX-treated group. In addition, SOD and GSH levels significantly increased in both the OLE and OLE+AgNP-treated groups compared to the DOX group.

Both OLE and OLE+AgNP, particularly OLE+AgNP, ameliorated DOX-induced testicular tissue injury, as evidenced by reduced injury and improved seminiferous tubules and spermatocyte area. In addition, OLE and OLE+AgNP, especially OLE+AgNP, inhibited DOX-induced testicular tissue inflammation, apoptosis, and endoplasmic reticulum stress.

The findings suggest that nanotechnology and the production of OLE+AgNP can ameliorate DOX-induced testicular damage.

**Graphical abstract:**

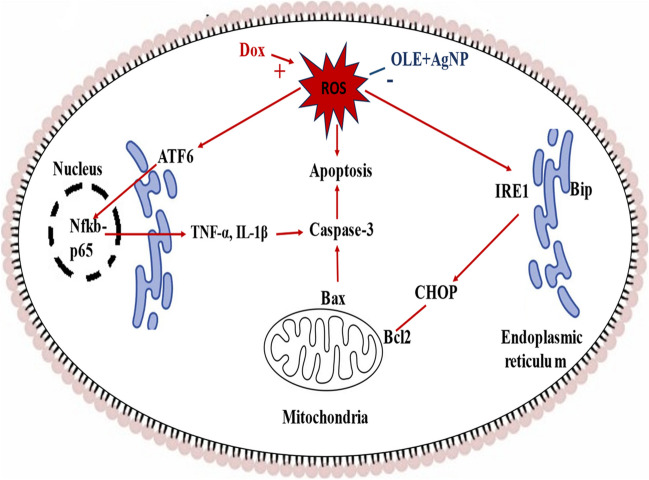

## Introduction

Cancer is a major health problem worldwide. The number of people diagnosed with cancer is increasing day by day. Various chemotherapeutic drugs are used in cancer treatments. However, the use of these drugs causes severe side effects in tissues and organs. Therefore, adjunctive agents are needed to minimize the side effects and organ damage caused by chemotherapeutic drugs. One commonly used chemotherapeutics is Doxorubicin [1]. Doxorubicin (DOX, Adriamycin) is an antibiotic from the anthracycline group used in cancer treatment [2, 3]. It is frequently used to treat sarcoma, carcinoma, and hematological cancer types. However, its use in chemotherapy is limited due to its cardiac, pulmonary, hepatic, renal, hematological, and testicular toxicity [4–7]. In studies using Doxorubicin, significant decreases in testis, epididymis, seminal gland, and prostate weights and sperm count, motility, and testosterone levels in rats were observed, significant increases in sperm abnormality, decrease in seminiferous tubule diameters and germinal cell layer thickness, impaired spermatogenesis, degeneration, edema, and necrosis were observed, and also severe testicular histopathological lesions [8–11]. It is also reported that it causes significant changes in the levels of both long (C18-C22) and very long (C24-C32) chain unsaturated fatty acids, which are in the testicular tissue of rats and participate in the structure of the sperm plasma membrane [12]. In mice, Doxorubicin causes significant decreases in normal sperm morphology, daily sperm production, and Sertoli cell number, dysregulation of mRNA levels of Sertoli and germ cells-related genes [13], histopathological lesions, such as a decrease in testicular weight, narrowing of seminiferous tubule diameters, and damage to spermatogenesis. It has been reported to cause [14], testicular DNA damage [15], chromosomal aberrations in spermatogonia [16], and apoptosis [17].

As a result of various recent studies, it has been stated that AgNPs have antioxidant and anti-inflammatory effects [18]. Additionally, neuropathy in diabetic rats [19], dextran sodium sulfate-induced colitis in mice [20], streptozotocin-induced hepatotoxicity in rats [21], diethyl nitrosamine-induced hepatocarcinogenesis in mice [22], alloxan-induced diabetes in mice [23], and collagen-induced rheumatoid arthritis in mice [24]. AgNPs have been reported to have promising effects in treating various inflammatory diseases due to their antioxidant and anti-inflammatory effects.

Recently, the number of studies using plant-derived compounds to minimize the cytotoxic effects of antineoplastic drugs or treat the resulting damage has been increasing [25]. One of the compounds used for this purpose is OLE. OLE, which is the building block of *Olea europea* (Olive tree) from the *Oleoceace* family, consists of polyphenols, alkanoic acids, and secoiridoids [26, 27]. Although it is also found in the Gentianacea (St. John’s Wort) and cornoleae families, OLE is also found in many other plants [28]. OLE, the main phenolic compound in olive leaves, is also found in olive oil and its fruit, but its main source is known as olive leaves.

In recent years, some nanoparticle loadings have been made to increase the effects of more stable substances and the effects of these substances on drug interactions and intracellular transitions. Among the nanoparticles loaded into natural components, silver nanoparticles hold an important place. In line with this information, in this study, we aimed to evaluate the protective effects of OLE loaded with silver nanoparticles on male reproductive system function against testicular tissue damage caused by Doxorubicin in rats.

## Materials and Methods

### Chemicals

Oleuropein (OLE), Silver nitrate (AgNO_3_), Sigma Chemical Co. (St. Louis, MO, USA), IL-1β antibody (sc-52012, Santa Cruz), TNF-α antibody (sc-52746, Santa Cruz), Caspase-3 antibody (sc-56053, Santa Cruz), Bcl-2 antibody (sc-7382, Santa Cruz), Nf-kB-p65 antibody (sc-109, Santa Cruz), CHOP antibody (AF6277, Affinity Biotec), ATF6 antibody (DF6009, Affinity Biotec), IRE1 antibody (DF7709, Affinity Biotec) and Beta-actin antibody (sc-47778, Santa Cruz) were obtained.

### Animal

The protocol of this study was approved by the Ataturk University Animal Experiments and Local Ethics Committee (2023/123). In the experimental study, 48 Sprague-Dawley adult male rats (12 weeks old) obtained from Atatürk University Experimental Research Center were divided into eight equal groups (n: 6). Rats were fed in separate cages containing three rats each and housed at an ambient temperature of 22 (±2)°C. The light pattern was adjusted to provide a 12-h light and 12-h dark cycle. Rats in all groups were allowed free access to water and food (Bayramoglu Feed and Flour Industry Trade A.Ş., Erzurum).

### Synthesis and Characterization of Citrate-Coated Silver Nanoparticles (AgNP)

For the synthesis of AgNP, 0.0167 g of silver nitrate (AgNO_3_) was dissolved in 100 ml of distilled water. Then, 0.020 g sodium citrate (Na_3_C_6_H_5_O_7_) (It is used as a reductant and stabilizer.) was dissolved in 20 ml distilled water in a flask. AgNO_3_ was allowed to stir until boiling. 5 ml Na_3_C_6_H_5_O_7_ was added dropwise to the boiling AgNO_3_ solution and boiled for about 1 h in a heated magnetic stirrer until a color change was observed. As a color change, the color of the mixture changed from light yellow to dark brown. This showed that Ag^+^ was reduced to Ag^0^. The formation of silver nanoparticles was also confirmed by the UV-Vis spectroscopy approximately at 427 nm. Finally, it was allowed to cool at room temperature and stored ready for use [29, 30].

In this study, oleuropein molecule was interacted with negatively charged synthesized citrate-coated silver nanoparticles (AgNP). For this purpose, oleuropein was thoroughly stirred in the synthesized AgNP solution for 3 h. As a result, stable structures containing citrate-coated silver nanoparticles were formed electrostatically to oleuropein molecules [31].

Characterization of AgNP was observed using ultraviolet-visible spectroscopy (UV-Vis) (a Perkin Elmer Lambda 35 spectrophotometer), which operated within the wavelength range of 200 to 800 nm. Functional groups were examined using Fourier-transform infrared (FT-IR) spectra were recorded using a PerkinElmer Frontier FT-IR spectrophotometer, operating within a wavelength range of 400 to 4000 cm^−1^. Transmission electron microscopy (TEM) analysis was done by Hitachi model HighTech-7700. Scanning Electron Microscopic (SEM) analysis was done by FEI QUANTA model FEG 450. Thin films of the nanoparticle sample were prepared on a carbon-coated belt, a very small amount of sample was placed on the sample holder and the excess solution was removed using a blotter, then the film on the SEM was allowed to dry under a mercury lamp for a few minutes.

### Experimental Design

The rats were randomly divided into eight groups, with six rats in each group, considering their live weight. The groups to be formed within the scope of this study and the experimental applications to be performed on the rats in each group are listed in Table 1 below.

**Table 1:** Groups and applications

Group name	Application
Control	Rats in this group were administered 0.5 ml of distilled water by gavage for 11 days.
OLE	The rats in this group were given Oleuropein at a dose of 50 mg/kg by gavage for 11 days.
AgNP	Silver Nanoparticle was given to the rats in this group by gavage at a dose of 100 mg/kg for 11 days.
OLE-AgNP	Oleuropein (50 mg/kg) loaded with Silver Nanoparticles was given to the rats in this group by gavage for 11 days.
DOX	The rats in this group were given Doxorubicin at a dose of 2.5 mg/kg i.p., once every 2 days, for a total dose of 15 mg/kg, 6 times in total.
OLE+DOX	Rats in this group were given Doxorubicin+Oleuropein: 2.5 mg/kg i.p. every other day + Oleuropein 50 mg/kg by gavage for 11 days.
AgNP+DOX	Rats in this group were given Doxorubicin+Silver Nanoparticle: 2.5 mg/kg i.p. every 2 days + Silver Nanoparticle 100 mg/kg via gavage for 11 days.
OLE-AgNP+DOX	Rats in this group were given Doxorubicin+Silver Nanoparticle + Oleuropein: 2.5 mg/kg i.p. every 2 days + Oleuropein loaded with Silver Nanoparticle (50 mg/kg) by gavage for 11 days.

### Sperm Analysis

It was carried out to determine the effect of Oleuropein-loaded silver nanoparticles on doxorubicin-induced changes in sperm parameters in testicular tissue samples obtained from experimental groups. Testis tissue from rats was separated from epididymis and both tissues were weighed separately. The cauda epididymis was kept in a petri dish in 5 ml of physiological serum at a temperature of 35°C for 5 min to migrate sperm cells. For sperm motility, 20 μl of semen was dropped onto the slide and covered with a slide. Scoring was done by selecting three different fields in a light microscope (Zeiss Primo Star, Carl Zeiss, Oberkochen, Germany) equipped with a heating plate. For sperm density, 10 μl of semen sample was mixed with 990 μl of physiological serum and vortexed. Then, a count was made on the Thoma slide. The result was determined by multiplying the found value by 5 × 10^6^. For the percentage of dead sperm and sperm abnormalities, 10 μl of semen and 10 μl of eosin dye (5%) were dripped and dried on a slide and mixed with a slide, and the slide was evaluated under a light microscope. Two hundred sperm cells were examined in each case. The sperm head–stained cells were considered dead. Abnormalities of sperm cells were determined in the same slides.

### Oxidative Stress Parameters Analysis

It was carried out to determine the effect of Oleuropein-loaded silver nanoparticles on doxorubicin-induced testicular tissue oxidative stress in testicular tissue samples obtained from experimental groups. Testicular tissue samples washed with PBS were lysed with Qiagen Tissue Lyser II at 30 Hz for 3 min by adding liquid nitrogen. Then, 0.1 g of tissue sample was added to homogenate buffers and homogenized in Tissue Lyser II at 30 Hz for 30 s.

#### SOD Enzyme Activity Analysis

The method is based on the principle of superoxide dismutase enzyme inhibiting free radicals during the reduction of free oxygen radicals released by enzymatic reaction in the presence of Nitrobluetetrazolium (NBT) in the sample. The color change observed due to the reaction was measured with a spectrophotometer at 560 nm [32].

#### MDA Level Analysis

LPO measurement was performed according to Ohkawa et al. [33] and was based on the principle of measuring the color formed by MDA with TBA in an acidic environment at 532 nm. Homogenate Preparation: By adding 2.5 ml 10% KCl to the tissue ground with 25 mg nitrogen, the mixtures were homogenized with the Tissue Lyzer LT homogenizer at 30 Hz for 3 min. Homogenates were centrifuged at 4000 rpm, 4°C for 30 min, and absorbance values of these supernatants were read against blank at 532 nm and tissue MDA concentration was calculated using the formula: Tissue MDA (μmol/mg tissue): Determination of malondialdehyde (MDA) is based on the principle of measuring the absorbance of the pink compound formed by the reaction of thiobarbituric acid (TBA) and MDA at a wavelength of 532 nm [33].

#### GSH Level Analysis

GSH levels in the testicular tissues of rats were determined by spectrophotometric method according to Sedlak et al. [34]. The principle of the method is that the color intensity of dark yellow 5-thio2-nitrobenzoic acid (TNB), which is released by the reduction of Ellman’s reagent (5,5′-dithiobis (2-nitrobenzoic acid); DTNB), by free thiol groups, is 412 nm. It is based on measuring wavelength (2 GSH + DTNB→ G–S –S–G + 2 TNB (dark yellow color)). Homogenate buffer: 50 mM pH 7.4, Tris-HCl (1.514 g Tris was dissolved in 200 ml of pure water, pH 7.4 was made and the final volume was completed to 250 ml with pure water). The obtained values were evaluated by comparing between groups.

### Histopathological Analysis

It was carried out to determine the effect of Oleuropein-loaded silver nanoparticles on doxorubicin-induced testicular tissue damage in testicular tissue samples obtained from experimental groups. Rat testicular tissues obtained at the end of the experiment were placed in 10% neutral formaldehyde solution and fixed for 72 h. Then, it was passed through graded alcohol and xylene series, embedded in paraffin blocks, and 5μ thick serial sections were taken with a microtome device (Leica RM2125 RTS) for histopathological evaluations. For histopathological examination, the sections were stained with Crossman’s Modified Mallory’s Triple Stain method, and tissue damage was evaluated. Johnsen’s mean testicular, histological damage and spermatogenesis in testicular tissue were evaluated under light microscopy. Thirty tubules for each testicle were graded according to the presence or absence of germ cell types, such as spermatozoa, spermatids, spermatocyte, spermatogonia, germ cells, and Sertoli cells in the testicular seminiferous tubules, and a score from 1 to 10 was given for each tubule. A higher Johnsen score indicates better spermatogenesis, while a lower score indicates severe dysfunction. A score of 1 means that no epithelial maturation is considered for tubules that are completely immobile; a score of 10 indicates that full epithelial maturation is considered for tubules with maximum activity [35]. A trinocular microscope with a computer and camera attachment (Zeiss AXIO Scope.A1, German) was used for microscopic examination. Histopathological evaluations were carried out according to the criteria specified in Tables 2 and 3.

**Table 2:** Histological classification of seminiferous tubular sections according to the Johnsen scoring system.

Score: Description	
10: Complete spermatogenesis is observed with many spermatozoa. The germinal epithelium has a clear lümen and the epithelium is of regular thickness.	
9: Many spermatozoa are present, but the germinal epithelium appears disorganized with marked sloughing or obliteration of the lumen.	
8: There are only a few sperm in the section.	
7: There are no spermatozoa, but there are many spermatids.	
6: No spermatozoa and only a few spermatids are present.	
5: There are no spermatozoa and no spermatids, but there are a few or many spermatocytes.	
4: There are only a few spermatocytes and no spermatids or spermatozoa.	
3: Spermatogonium is the only germ cell present.	
2: There are no germ cells, but there are Sertoli cells.	
1: There are no cells in the tubular section.

**Table 3:** Testis weight, epididymis weight, sperm motility, abnormal sperm rate, dead sperm ratio, and density parameters in the experimental groups.

Experimental groups	Total testis weight (mg)	Total motility (%)	Density(×10^6^)	Live/dead spermatozoon rate (%)	Abnormal spermatozoon rate (%)	Total epididymis weight (mg)
Control	2634.20±320.93	74.01±4.18^b^	20.01±4.89^a^	8±0.7^a^	4.4±1.14^a^	370.40±39.22 ^abc^
OLE	2875.20±220.10	81.5±5.07^bc^	27.80±6.34 ^ab^	7±1.41^a^	3.4±0.54^a^	336.80±44.84 ^ab^
AgNP	2946.01±465.83	91.01±4.18^bd^	37.20±11.36^b^	7.20±0.83^a^	3.6±0.54^a^	457.40±24.13^c^
OLE-AgNP	3039.80±268.19	87.01±5.70^cd^	31.80±5.76^ab^	9.20±0.84^a^	3.6±0.54^a^	449.60±30.90^c^
DOX	3120.01±434.82	44.01±15.16^a^	27.60±5.02 ^ab^	23.60±4.50^b^	18.6±4.34^b^	313.01±103.31^a^
OLE+DOX	2857.61±205.73	66.01±4.18^cd^	27.01±6.09 ^ab^	23.40±3.39^b^	15.8±2.16 ^b^	394.40±56.85 ^abc^
AgNP+DOX	2574.60±99.67	80.01±3.53^cd^	26.41±6.51 ^a^	18.6±3.20^b^	16.40±2.60 ^b^	427.01±38.98^c^
OLE-AgNP+DOX	3066.01±116.49	86.60±7.26^cd^	30.61±3.20 ^ab^	10.2±2.28^a^	15.01±2.23 ^b^	410.20±38.14 ^abc^

Different lowercase letters (a, b, c, d; *P* <0.001) in the same line are statistically significant (*P*<0.001)

### Western Blot Analysis

It was performed to determine the protein levels of testicular tissue reticulum stress, apoptosis and inflammation markers in testicular tissue samples taken from the experimental groups. The testicular tissue samples taken were stored in a deep freezer at −80°C before western blot analysis. Testicular tissue samples were weighed and crushed in nitrogen gas, treated with radioimmunoprecipitation (RIPA buffer, Ecotech Bio, Turkey) supplemented with protease and phosphatase inhibitors, and homogenized for 20 s at 30 Hz using a tissue disruptor (Qiagen, USA). It determines the relative protein expressions of IL-1β, TNF-α, Caspase-3, Bcl-2, Nf-kB-p65, ATF6, and IRE1. A protein assay kit was used to measure the total protein of testicular tissue (Pierce BCA, Thermo Sci., USA). 30 μg of protein was then placed on the PVDF membrane after separation by 10% SDS-PAGE. First, membranes were blocked for 90 min at room temperature using 5% bovine serum albumin. The membranes were then incubated with appropriate primary antibodies overnight at 4°C. After primary antibody incubation, PVDF membranes were washed with TBST and then incubated with a secondary antibody conjugated to horseradish peroxidase (Santa Cruz, sc-2004/sc-2005) for an additional 90 min at room temperature. Protein bands were then captured using enhanced chemiluminescence reagent Western ECL substrate (Thermo, 3405), visualized, and analyzed by Image Lab™ Software (Bio-Rad, Hercules, CA, USA).

### Statistical Analysis

Statistical analysis was performed using the SPSS (version 25.0; IBM SPSS Inc, Chicago, IL, USA) package program. The normality of the data was determined by the Kolmogorov-Smirnov test. Descriptive statistical analyses (mean ± standard deviation) were used. One-way ANOVA test and post hoc Tukey test were applied to compare groups. *p*-values less than 0.05 at the 95% confidence interval were considered statistically significant.

## Results

### Characterization of Citrate-Coated Silver Nanoparticles (AgNP)

**Fig. 1:**
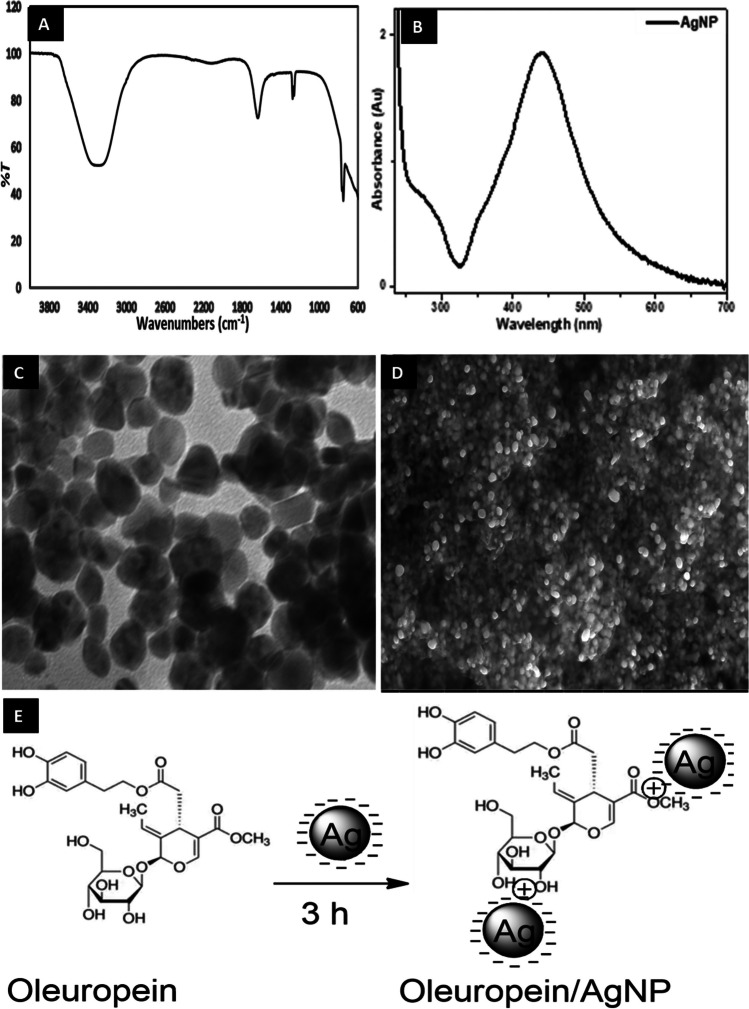
(**A**) The FT-IR spectrum of AgNP, (**B**) UV spectrum of citrate-coated AgNP, (**C**) The TEM spectrum of AgNP, (**D**) The SEM images of citrate-coated AgNP, (**E**) Chemical structure of oleuropein interacting with citrate-coated AgNP

As it is known, many methods are used to characterize nanoparticles. In this study, FT-IR, UV, TEM, and SEM spectroscopies were used for the characterization of AgNP. As a result of the FT-IR analysis, spectra at a wavelength of 3284 cm^−1^ show that it originates from O-H groups. Strong bands originating from -CH groups at a wavelength of 1639 cm^−1^, 1276 cm^−1^, 1261 cm^−1^, and 764 cm^−1^, 751 cm^−1^ show stretching vibrations in C=O groups and C=C also show stretching vibrations (Fig. 1A). The strong absorbance of the obtained AgNP at approximately 427 nm. This band is typical for AgNP and confirms the formation of AgNP. It is also in agreement with the literature (Fig. 1B) [36]. Also, the shape of AgNP was determined by TEM. The TEM image of synthesized citrate-coated AgNP is shown in Fig. 1C. The average size of citrate-coated AgNP was found to be about 10–60 nm and has a spherical morphology. Scanning electron microscopy (SEM) images of citrate-coated AgNP are shown in Fig. 1D. SEM analysis, in addition to size the distribution of the synthesized citrate-coated AgNP appears to be quite well dispersed, while the average size of the citrate-coated AgNP is about 10–56 nm.

For nanoparticles, the presence of negatively charged citrate molecules (which use sodium citrate as a reducing agent and stabilizer) has several important functions compared to other groups. First of all, it helps to achieve chemical stability by lowering the surface energy of highly active nanoparticles. Another property is to stabilize the nanoparticles, preventing their agglomeration and ensuring a good dispersion of the nanoparticles, which is necessary for their interaction with other molecules [37–39]. In this study, the oleuropein molecule interacted with negatively charged synthesized citrate-coated silver nanoparticles (AgNP) to electrostatically attach oleuropein molecules to stable structures containing citrate-coated silver nanoparticles (Fig. 1E).

### Sperm Analysis Results

Spermatological parameters and reproductive organ weights are presented in Table 2. There was no difference in testicular weight between the groups. While the total motility value was the lowest in the DOX group, OLE and nanoparticle treatment improved the total motility value. Spermatozoon density was generally higher in the AgNP-applied group (*P*<0.001). While the rate of dead and abnormal spermatozoa increased statistically in DOX-treated groups, AgNP and OLE treatment improved these parameters. Epididymis weight was higher in AgNP-applied groups than in other groups (*P*<0.001).

### Oxidative Stress Parameters

**Fig. 2:**
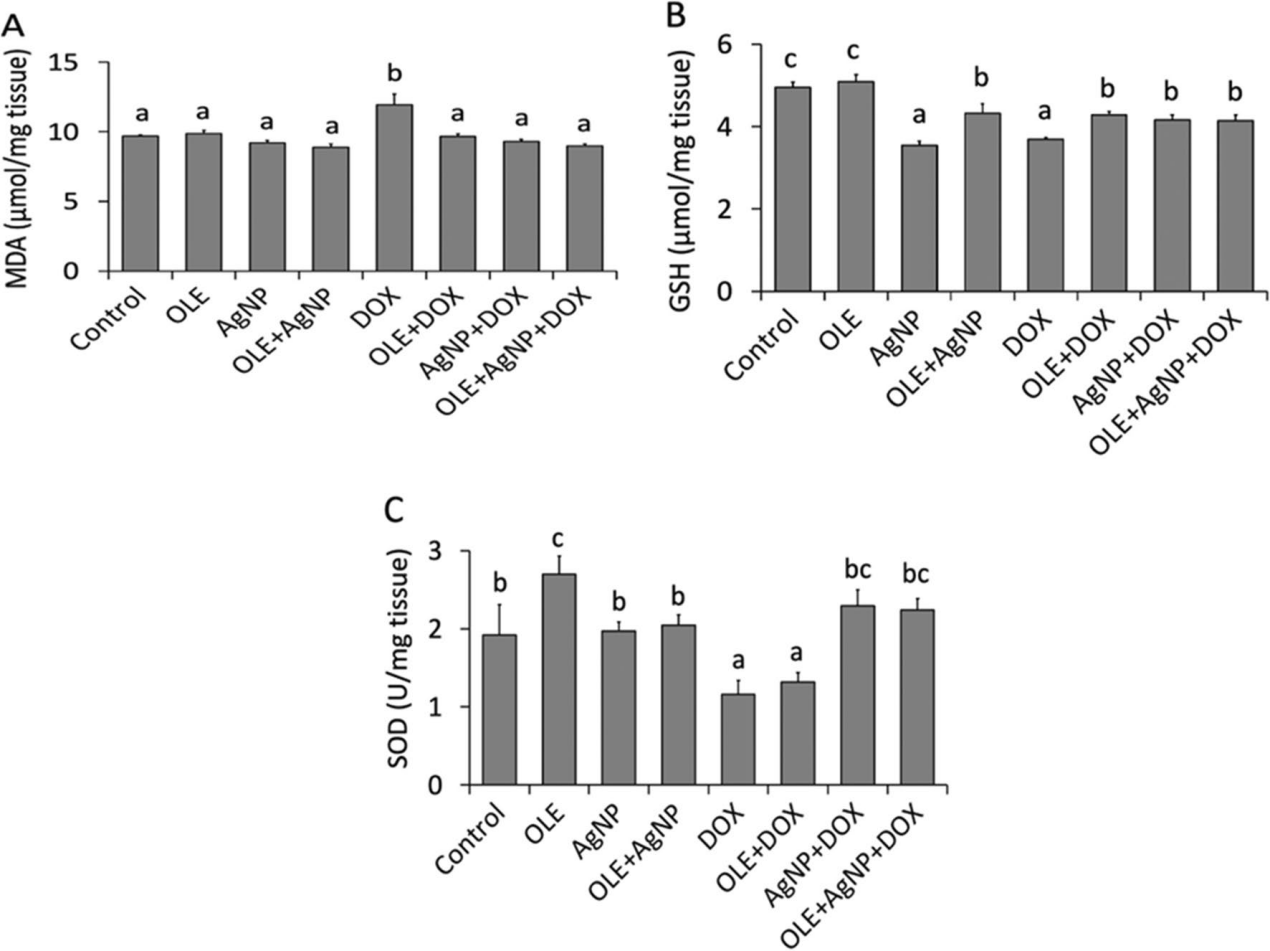
Biochemical tissue oxidant and antioxidant parameters of all groups. The values are given as mean ± SD (*n*=6) and analyzed by one-way ANOVA followed by the Tukey test. The letters (**a**,**b**,**c**) indicate statistically significant differences between the groups, *P*<0.05

Data analysis showed a notable decrease in MDA levels in both the OLE-receiving group (*P* < 0.05) and the OLE+AgNP group (*P* < 0.05) compared to the DOX group (Fig. 2). In addition, rats in the DOX group showed a notable decrease in SOD levels compared to the control, OLE, AgNP, OLE+AgNP, AgNP+DOX, and OLE+AgNP+DOX group (*P* < 0.05). Regarding GSH levels, we noticed a notable decrease in rats receiving DOX compared to the control group (*P* < 0.05). However, compared with the DOX group, the findings showed a significant increase in GSH levels in rats receiving OLE (*P* < 0.05) and OLE+AgNP (*P* < 0.05) (Fig. 2).

### Histopathologic Analysis Results

Seminiferous tubules were graded (1 to 10) according to the decrease in the number and density of germ cells from the lumen of the seminiferous tubules. According to Johnsen scores, the presence of a few too many spermatozoa in a part of the seminiferous tubules with scores of 8, 9, and 10 indicates spermatogenesis. If the score is 6 and 7, there are no spermatozoa in the seminiferous tubules, but spermatids are present. The score is evaluated as 4 and 5 in seminiferous tubules that do not contain spermatozoa and spermatids but do contain spermatocytes. The score is evaluated as 3 in seminiferous tubules that do not contain germ cells other than spermatogonia. Score 2 is given to tubules that do not contain germ cells but contain Sertoli cells. In seminiferous tubules with no cells, the score is evaluated as 1. In the current study, testicular tissue was observed to be normal in the Control, OLE, AgNP, and OLE-AgNP groups, and no pathology was observed in the seminiferous tubule epithelium. In the DOX group, testicular integrity is completely disrupted, and the seminiferous tubules lose their normal structure. It is observed that the germ cells within the seminiferous tubules are completely shed in some tubules, while in others, they display an irregular and incomplete appearance. In some seminiferous tubules, the lumen is observed to be completely obliterated.

**Fig. 3:**
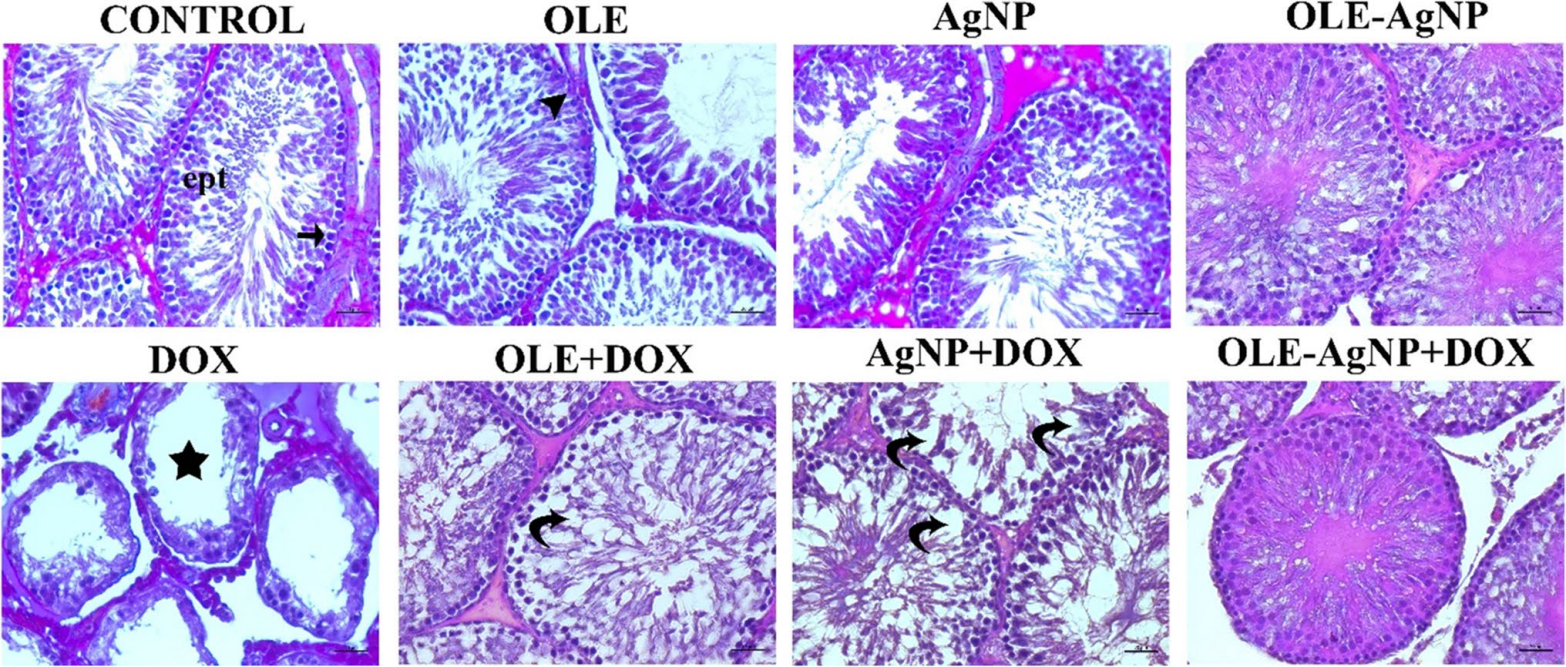
Testicular seminiferous tubules stained with Mallory’s Triple Stain Modified by Crossman. Star: Degeneration in seminiferous tubules, Arrow: Spermatogonia, Curved arrow: Deformation in tubule epithelium, Arrowhead: Sertoli cells. Magnification: 400×

**Fig. 4:**
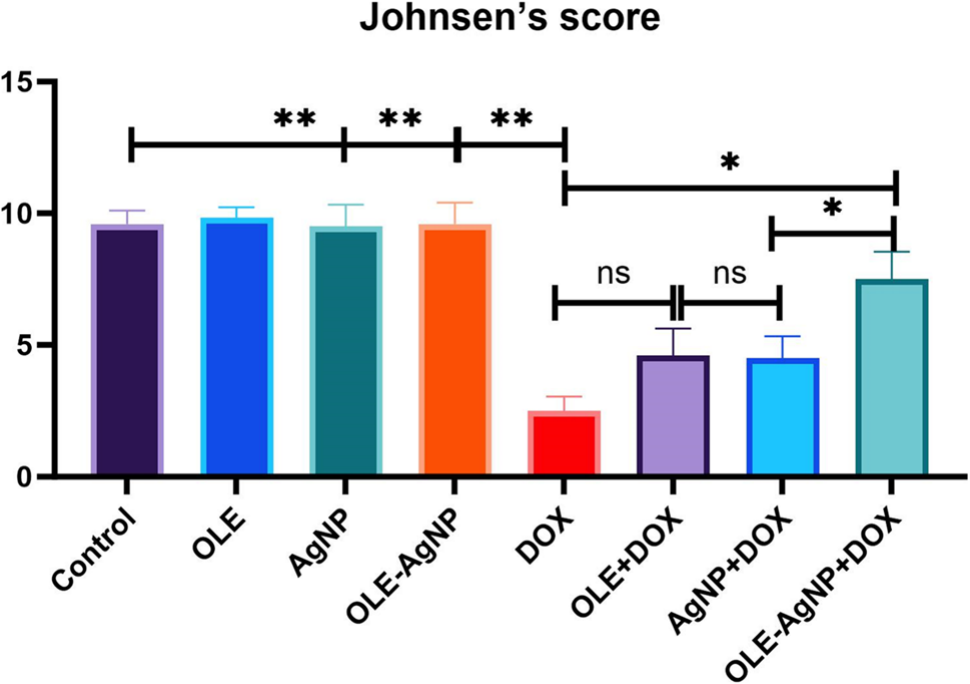
Assessment of testicular histology according to Johnsen’s scoring system. The values are given as mean ± SD (*n*=6) and analyzed by one-way ANOVA followed by the Tukey test. The asterisk (*: *p*<0.05; **: *p*<0.001; ns: not significant) indicates statistically significant differences between the groups, *p*<0.05

Degeneration in seminiferous tubules and germ cells is identified. It was observed that the integrity of the seminiferous tubules was maintained, the germ cells were arranged regularly, and the tubule lumens had a normal structure, especially in the OLE-AgNP+DOX group and in the OLE+DOX and AgNP+DOX groups. Accordingly, because of histopathological evaluations, it was observed that the score was 10 in the Control, OLE, AgNP, and OLE-AgNP groups according to the Johnsen scoring system and three in the DOX group. According to this scoring system, while the score was 5 in the AgNP+DOX and OLE+DOX groups, it was determined as 8 in the OLE-AgNP+DOX group (Figs. 3 and 4).

### Western Blot Analysis Results

**Fig. 5:**
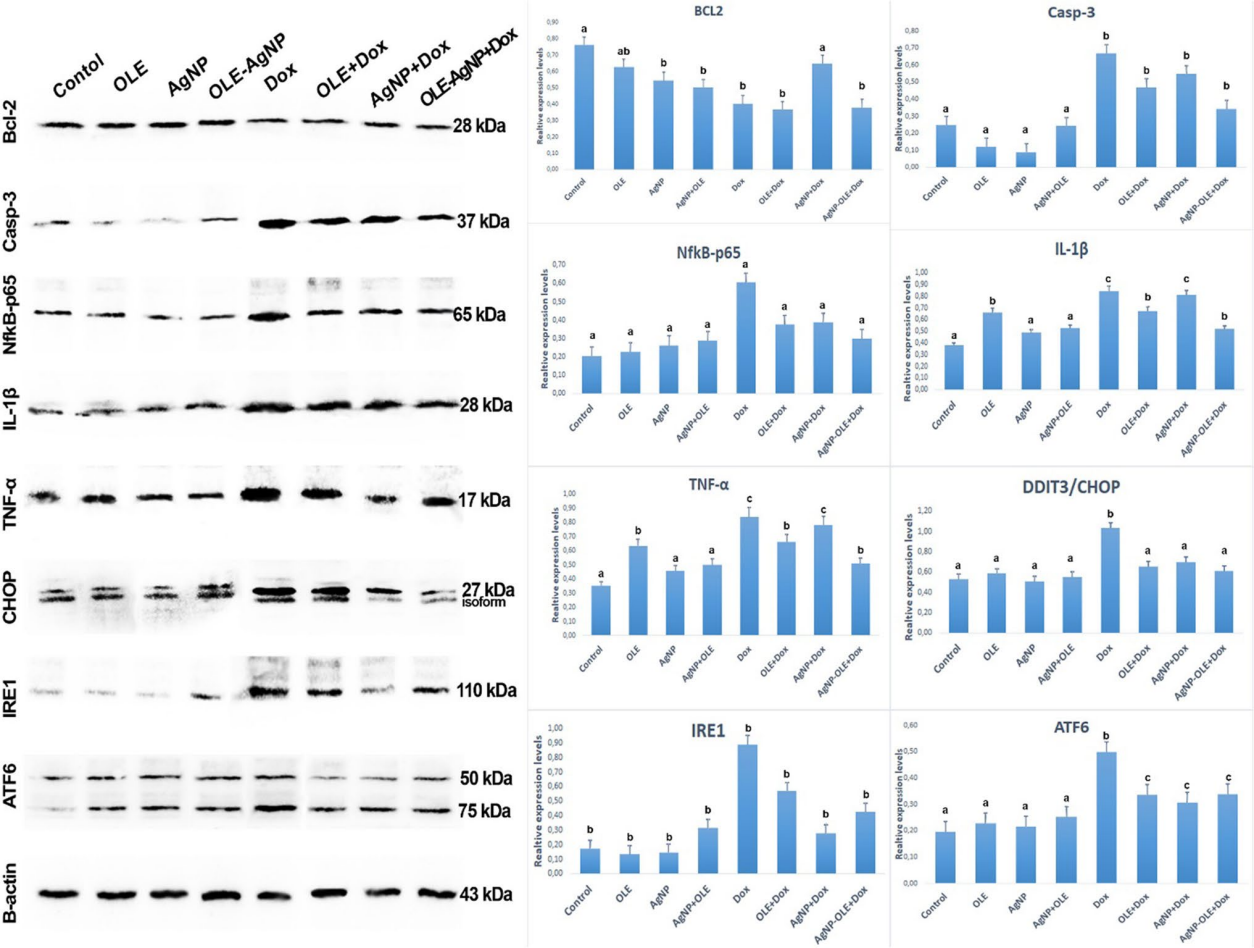
Relative expression of proteins for ATF6, IRE1, Bcl-2, TNF-α, IL-1B, CHOP, Caspase-3, Nfkb-p65. The values are given as mean ± SD (*n*=6) and analyzed by one-way ANOVA followed by the Tukey test. The letters (a,b,c) indicate statistically significant differences between the groups, *P*<0.05

In the evaluation of protein levels obtained from the study tissues, it was observed that the expression level of ATF6, NfkB-p65, and CHOP was significantly increased in the DOX group compared to the other groups (*p*<0.05). However, there was no statistical difference between the Control, OLE, AgNP, and OLE-AgNP groups (*p*>0.05). Again, there was no statistical difference in the OLE+DOX, AgNP+DOX, and OLE-AgNP+DOX groups (*p*>0.05). It was observed that there was a significant increase in the expressions of IRE1, TNF-α, Caspase-3, and IL-1β in the DOX group compared to the other groups (*p*<0.05). It was observed that this expression level decreased significantly in the OLE-AgNP+DOX group (*p*<0.05). This showed that silver nanoparticle-loaded OLE prevented the increase of IRE1, TNF-α, Caspase-3, and IL-1β in testicular tissue. Bcl-2 expression was lowest in the DOX, OLE+DOX, and OLE-AgNP+DOX groups, and there was no significant difference between them (*p*>0.05). Expression analysis and comparisons of all groups and proteins are presented in Fig. 5.

## Discussion

Most of the drugs used in cancer treatment prevent the growth and proliferation of malignant cells with their cytotoxic effects and cause them to die. Doxorubicin is an effective chemotherapeutic agent from the anthracycline group, which is used in cancer treatment and has many side effects [4]. It is most frequently used in hematological malignancies, sarcoma, lymphoma, prostate carcinoma, thyroid, lung, and breast carcinomas. The biggest concern during the use of chemotherapeutic drugs is their toxicity. Studies have shown that many chemotherapy drugs may cause damage to reproductive organs. Doxorubicin may cause damage to testicular tissue, such as significant lipid peroxidation in the heart, kidney, liver, ovary, and testicular tissues [40].

It is accepted that DOX changes DNA strands and gene expression of various enzymes at transcriptional or translational stages, either directly or indirectly through the free radicals it creates, thus causing changes in antioxidant enzyme activities [41, 42]. In some studies, free radicals held responsible for pathogenesis are superoxide, hydroxyl radicals, and nitric oxide (NO). It has also been shown that lipid peroxidation products, such as MDA induced by free radicals, contribute to the event and cause cellular damage by reducing antioxidant enzymes, such as catalase (CAT), glutathione (GSH), glutathione peroxidase (GSH-Px) and superoxide dismutase (SOD) [43, 44]. Findings that free radicals and antioxidant enzymes are involved in the pathogenesis of DOX-related toxicity have brought antioxidant treatment trials to the agenda [45, 46]. In our study, we determined that DOX application decreased the SOD and GSH levels in the testicular tissue, while the MDA level increased. On the other hand, we determined that OLE and OLE+AgNP applications prevented this increase. It has been observed that silver nanoparticle–loaded OLE suppresses oxidative stress more effectively.

Studies in rats have shown that DOX causes a decrease in sperm count and viability and increases abnormal sperm morphology [47]. In our study, consistent with the findings obtained in the previous study [47], it was observed that DOX application caused a decrease in sperm count and viability. Rather, it caused an increase in abnormal sperm morphology. When the groups treated with OLE and OLE + AgNP were examined, it was determined that OLE and its silver nanoparticle-loaded form increased sperm count and viability and reduced the rate of abnormal sperm. These findings suggest that OLE and OLE+AgNP positively affect sperm count and morphology by suppressing oxidative stress and inflammation caused by DOX in the testicles with their antioxidant and anti-inflammatory activity.

Inflammation is associated with many chronic diseases, especially cancer, diabetes, cardiovascular and neurological diseases. However, it has been reported that various transcription factors, such as AP-1, NFĸB, and p53, are activated in case of oxidative stress [48, 49]. DOX induces inflammation by increasing pro-inflammatory cytokine levels and decreasing anti-inflammatory cytokine levels [50]. OLE has been experimentally found to prevent/reduce inflammation [51]. In this study, the findings showed that DOX application increased the expression of NfkB-p65, IL-1β, and TNF-α, thus increasing inflammation, while OLE and OLE+AgNP application suppressed the expression of NfkB-p65, IL-1β, and TNF-α.

ER is an organelle that is highly sensitive to intracellular and extracellular stimuli [52, 53]. When ER stress is activated for any reason, the amount of unfolded or misfolded protein in the cell increases, and accordingly, three transmembrane proteins called p-PERK, IRE1α, and ATF6, which combine unfolded proteins, are separated from GRP78 [54, 55]. Experimentally, ER stress can be induced in various tissues by DOX in rats [56], and when ER stress occurs, the expression levels of GRP78, CHOP, ATF6, p-IRE1, sXBP1, and p-PERK increase. According to the data obtained in our study, the DOX application increased the expression of CHOP, ATF6, and p-IRE1 in testicular tissue. Studies have shown that OLE application suppresses endoplasmic reticulum stress [57, 58]. In our study, we determined that OLE and OLE+AgNP reduced the expression of CHOP, ATF6, and p-IRE1 in testicular tissue in rats.

Some previous studies have observed that DOX application causes congestion, interstitial edema in rat testicles, and degeneration, necrosis, and calcification in spermatogenic cells in the seminiferous tubules [47]. It has also been reported that DOX reduces the levels of testosterone produced by suppressing the expression of key transcription factors and genes involved in the steroidogenic pathway in Leydig cells [59]. In our study, DOX treatment caused the integrity of the testicle to be completely disrupted in the testicular tissue, the seminiferous tubules lost their normal structure, the germ cells in the seminiferous tubules were completely shed in some tubules and caused an irregular and incomplete appearance in others, and OLE and OLE+AgNP caused these changes due to DOX.

Some recent studies have reported that ROS has a crucial role in stimulating the apoptotic mechanism in the cell [60, 61]. It occurs in apoptotic mechanisms through a pathway induced by members of the Bcl-2 protein family, which includes Caspase-3 and Bcl-2 [62–66]. Ujah et al. reported that in DOX-induced testicular toxicity in rats, Caspese-3 expression increased, and Bcl-2 expression decreased in the toxicity group compared to other groups [50]. In our study, DOX significantly increased Caspase-3 expression in a testicular tissue, whereas Bcl-2 expression decreased in a testicular tissue. On the other hand, it has been reported that OLE and silver nanoparticles suppress apoptosis in various tissues by decreasing Caspese-3 expression and increasing Bcl-2 expression. We determined that both OLE and silver nanoparticle–loaded OLE decreased Caspese-3 expression and increased Bcl-2 expression.

In conclusion, the findings obtained in this study indicate that DOX treatment triggers ROS production, thus stimulating ROS-mediated direct apoptosis, and triggering endoplasmic reticulum stress by ROS causes the suppression of mitochondrial Bcl2 and increased Bax production, thus increasing Bax causes apoptosis by stimulating caspase-3 formation. Again, increasing ROS stimulates NF-kB formation in the nucleus by triggering endoplasmic reticulum stress, thus increasing cytokine levels stimulate apoptosis. However, we determined that the OLE-AgNP application blocked these signaling pathways stimulated by DOX. These findings raised the possibility that OLE-AgNP or OLE could be an adjuvant therapy that protects testicular tissue from DOX-dependent oxidative and apoptotic effects and reduces its side effects.

## Data Availability

Data are contained within the article or supplementary material.
